# Drug-Induced Gingival Overgrowth Associated With Cyclosporine Therapy: A Case Report of a 23-Year Periodontal Follow-Up in a Heart Transplant Recipient

**DOI:** 10.7759/cureus.97019

**Published:** 2025-11-16

**Authors:** Pierluigi Valente, Lapo Sbrenna, Francesco Valente, Andrea Sbrenna, Andrea Mascolo

**Affiliations:** 1 Dentistry, School of Dentistry, Vita-Salute San Raffaele University, Milan, ITA; 2 Dentistry, School of Dentistry, Alma Mater Studiorum University of Bologna, Bologna, ITA; 3 Oral Implantology, San Damiano Dental Clinic, Rome, ITA; 4 Private Practice, Humanis Dental Center, Perugia, ITA; 5 Academics, European Institute for Medical Studies, St. Julian's, MLT

**Keywords:** cardiac transplant, cyclosporine, drug-induced gingival overgrowth (digo), gingivitis, heart transplant patient, immunosuppressive therapy, metagenomic sequencing, periodontal maintenance, periodontitis, porphyromonas gingivalis

## Abstract

Drug-induced gingival overgrowth (DIGO) is a common adverse effect of cyclosporine therapy, which is widely used as an immunosuppressive agent in solid organ transplant recipients. This case report describes the 23-year follow-up of a male patient with a history of orthotopic heart transplantation, performed two years prior to his first dental visit in 2002, who developed DIGO under long-term cyclosporine therapy. At the initial periodontal evaluation, a diagnosis of localized Stage I, Grade A periodontitis associated with gingivitis was made, and nonsurgical mechanical debridement, scaling and root planing, and tailored oral hygiene instruction were provided, yielding favorable early outcomes and long-term periodontal stability. After many years of stability, the patient returned following a three-year lapse in maintenance, presenting with marked gingival enlargement, bleeding on probing, discoloration, and migration of the maxillary central incisors, consistent with progression to Stage II, Grade B periodontitis. Nonsurgical retreatment was performed, and DNA-based metagenomic analysis of subgingival plaque and tongue biofilm revealed a dysbiotic microbial profile, including the persistence of key periodontopathogenic taxa associated with tissue destruction and alveolar bone loss. This case underscores the importance of sustained periodontal maintenance in transplant recipients receiving cyclosporine therapy and illustrates that even after decades of apparent stability, DIGO and periodontal deterioration may reemerge if maintenance care is interrupted. The integration of DNA-based metagenomic analysis provided valuable diagnostic and motivational support, reinforcing a personalized, multidisciplinary approach to long-term periodontal management in immunosuppressed patients.

## Introduction

Heart transplantation is a well-established treatment for end-stage cardiac disease, significantly improving patient survival and quality of life. However, lifelong immunosuppressive therapy, most commonly involving calcineurin inhibitors like cyclosporine, is necessary to prevent graft rejection and is associated with a range of systemic and oral side effects [1]. Among these, drug-induced gingival overgrowth (DIGO) is a frequently reported complication. DIGO has been shown to increase significantly during cyclosporine therapy, even in the context of sufficient oral hygiene and reduced gingival inflammation. In studies involving structured oral hygiene instruction and motivation programs, plaque and gingivitis levels declined markedly, yet gingival enlargement continued to progress. These findings suggest that cyclosporin blood concentration is the principal determinant of DIGO severity, with plaque accumulation and gingival inflammation serving as secondary contributing factors [2].

As early as 1994, Italian investigators had already documented the prevalence of this condition among transplant recipients. In a cohort of 19 patients undergoing cyclosporine A therapy, gingival overgrowth was observed in 88% of patients, affecting 35.9% of evaluated gingival sites [3]. DIGO typically presents as a lobulated, fibrotic enlargement of the gingiva, which may interfere with aesthetics, speech, mastication, and oral hygiene [4]. Histopathological features include connective tissue hyperplasia and epithelial hyperkeratosis, often accompanied by secondary fungal colonization such as candidiasis, particularly in immunosuppressed individuals [5]. The etiology of DIGO is multifactorial, involving drug effects, local plaque accumulation, individual susceptibility, and altered immune responses. Among the proposed mechanisms, transforming growth factor β1 (TGF-β1) has been implicated for its role in stimulating fibroblast activity and extracellular matrix production. Elevated gingival levels of TGF-β1 have been detected in cyclosporine-treated transplant recipients and correlate with systemic drug concentration [6].

In addition to gingival enlargement, oral complications in cardiac transplant patients include increased risk of periodontitis, fungal infections, and, over time, malignant lesions. A review by Gruter et al. reported higher rates of gingival inflammation, oral candidiasis, and a 4.3-fold increased risk of oral cancer in this population compared to healthy controls [7]. Management of DIGO in transplant recipients requires a multimodal approach that combines professional hygiene measures, control of local irritants, and, when necessary, surgical removal of hyperplastic tissues. Although switching to alternative immunosuppressive agents such as tacrolimus may yield clinical improvement [8], this option is not always feasible or medically indicated.

This case-based review illustrates the periodontal management of a heart transplant recipient who developed cyclosporine-induced gingival overgrowth over a 23-year dental follow-up period. Following a prolonged absence from maintenance visits, the patient returned with significant worsening of periodontal conditions, including clinical signs of progressive inflammation, gingival enlargement, discoloration, and tooth migration. In response to this deterioration, a multimodal treatment approach was adopted, combining conventional periodontal therapy with adjunctive microbiological assessments. DNA-based analysis of tongue biofilm and subgingival plaque from inflamed sites was performed to better understand the microbial environment and help arrest the progression of periodontal disease. The case is discussed in light of the current literature, offering practical insights for dental management of immunosuppressed patients, in the hope that its detailed description will help prevent similar year-by-year deterioration in comparable clinical scenarios.

This case provides a unique 23-year longitudinal perspective on the management of cyclosporine-associated DIGO, emphasizing the clinical consequences of interrupted periodontal maintenance and the potential role of modern metagenomic assessment in long-term patient care. This case report is based on routine clinical documentation and observational findings and does not constitute research involving human subjects under current institutional and regulatory definitions. In accordance with institutional policy, the case was deemed exempt from formal IRB/Ethics Committee review. Written informed consent was obtained from the patient for the dental treatment and for publication of this report and any accompanying images. The images included do not contain any data that could lead to patient identification.

## Case presentation

A 42-year-old Caucasian male, employed as an office worker, was first examined at the San Damiano Dental Center in Rome, Italy, in early 2002. The patient sought dental evaluation due to an inadequate prosthetic crown on the upper right central incisor. His medical history included an orthotopic heart transplantation performed in late 1999 for end-stage dilated cardiomyopathy. Since the transplant, he had been maintained on lifelong immunosuppressive therapy, consisting of cyclosporine (self-reported daily dose of 50 mg + 30 mg), in combination with mycophenolate mofetil and bisoprolol. No calcium channel blockers, such as amlodipine or nifedipine, known to exacerbate gingival enlargement, were included in his regimen. The patient denied tobacco use, alcohol consumption, or recreational drug use.

**Figure 1:**
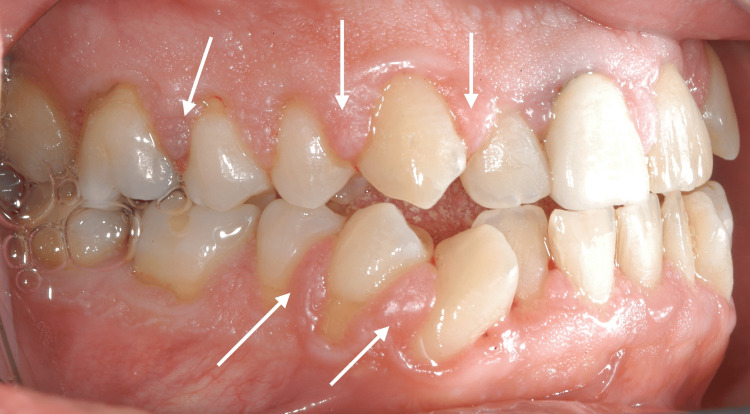
Intraoral photography from the patient's initial clinical examination in 2002, showing the right maxillary and mandibular hemi-arches with marked hypertrophy of the interdental papillae (white arrows).

**Figure 2:**
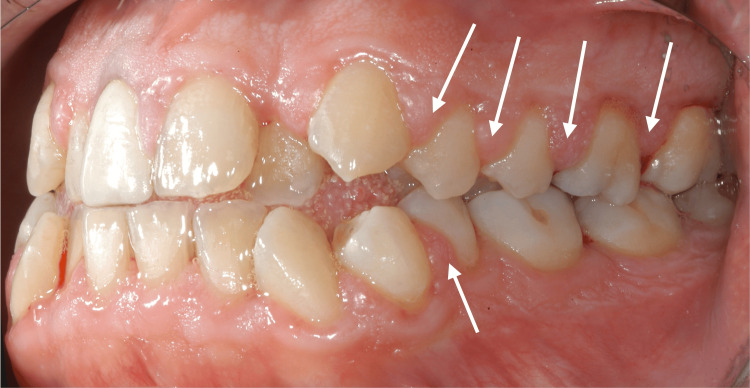
Intraoral photography from the patient's initial clinical examination in 2002, showing the left maxillary and mandibular hemi-arches with marked hypertrophy of the interdental papillae (white arrows).

**Figure 3:**
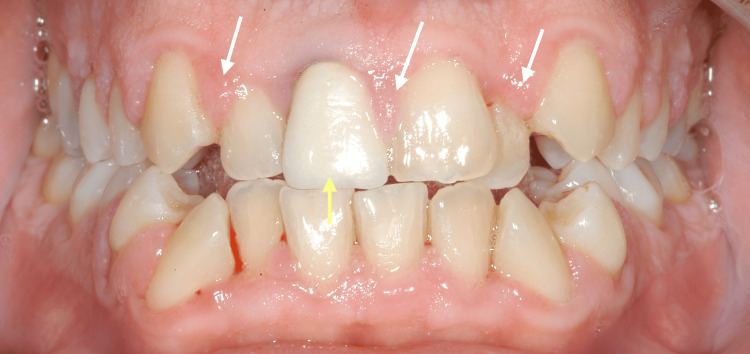
Intraoral frontal view from the patient's initial clinical examination in 2002, showing hypertrophic interdental papillae (white arrows) and the presence of an unaesthetic prosthetic crown on the upper right central incisor (yellow arrow), contributing to the compromised gingival architecture and an overall unaesthetic smile.

At the initial dental examination, the patient presented with generalized gingival overgrowth characterized by lobulated, erythematous tissue, most prominent in the right (Figure 1) and left (Figure 2) posterior regions. Hypertrophic interdental papillae were also evident in the anterior maxillary area (Figure 3).

Periodontal charting revealed probing depths of 4-5 mm at multiple localized sites, with bleeding on probing and evident plaque accumulation. Although gingival overgrowth created pseudo-pockets, true periodontal defects with 1-2 mm attachment loss were detected in some areas. Plaque Index and Bleeding on Probing were both elevated at baseline, reflecting poor plaque control secondary to the fibrotic gingival enlargement. The clinical condition was consistent with DIGO, most likely related to cyclosporine use, in association with gingivitis and localized initial periodontitis. Given the extensive longitudinal follow-up, the diagnosis was retrospectively expressed according to the 2018 Classification of Periodontal Diseases by the European Federation of Periodontology and the American Academy of Periodontology, corresponding to Stage I, Grade A localized periodontitis. This retrospective application was intended solely to ensure terminological consistency with current periodontal standards and does not imply that this classification was available at the time of the initial diagnosis [9].

**Figure 4:**
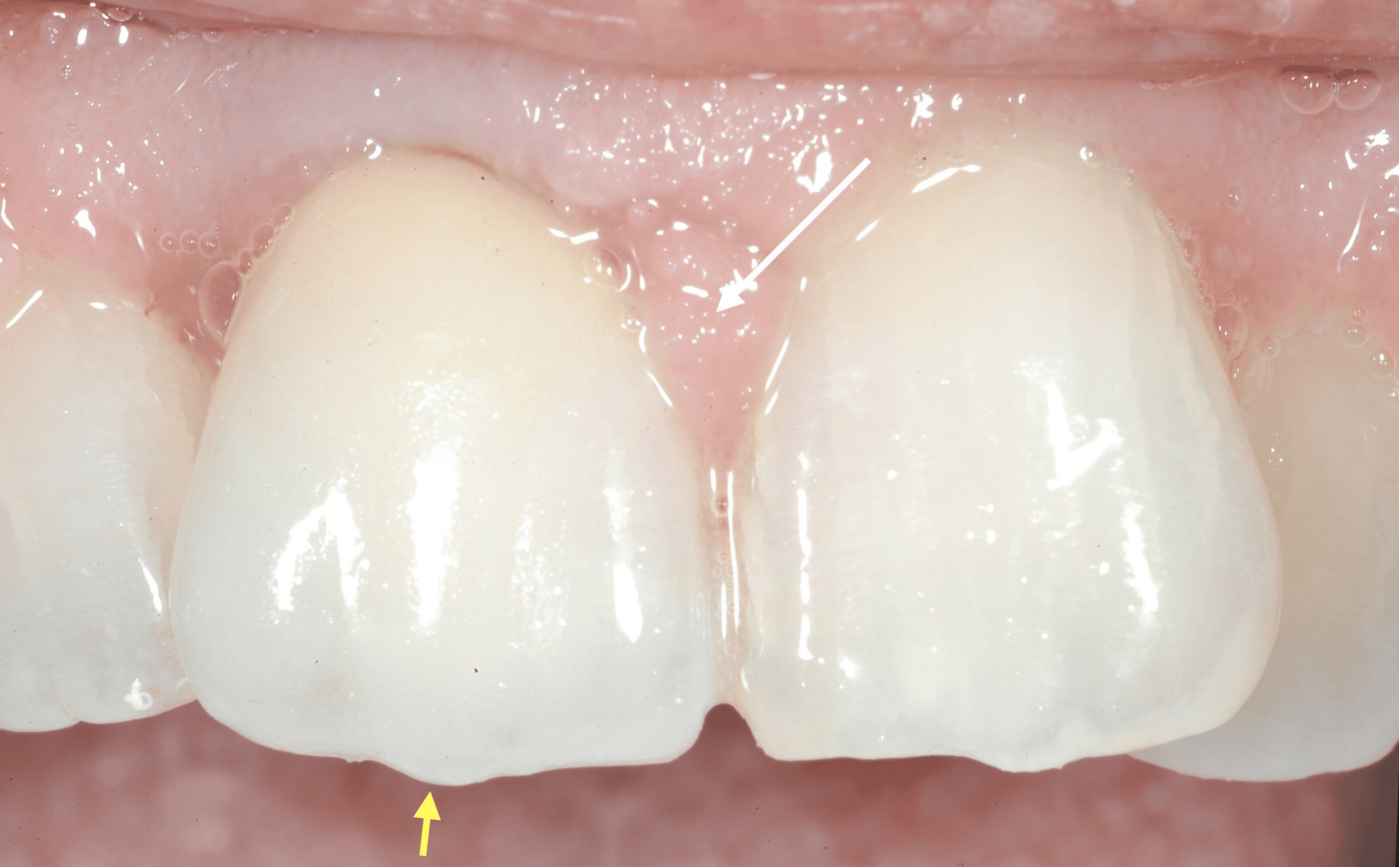
An intraoral view captured a few months after the patient's initial clinical examination in 2002, showing the newly restored maxillary right central incisor (yellow arrow) and the hypertrophic interdental papilla between the two central incisors (white arrow), still present despite the patient's hygiene efforts and regular follow-up appointments.

In line with standard protocol, the treatment approach included an initial Phase I periodontal therapy, consisting of professional oral hygiene instructions, supragingival and subgingival biofilm removal, and motivation. Concurrently, the inadequate prosthetic crown was replaced with a properly contoured restoration to eliminate local contributing factors (Figure 4).

Following a comprehensive periodontal intervention in 2002, the patient remained under maintenance care for more than two decades. Both clinical charting and full-mouth radiographic evaluations were performed at regular intervals, consistently confirming long-term periodontal stability. Initially, adherence to oral hygiene instructions and supportive periodontal therapy was excellent; however, over time, compliance gradually declined, leading to increasingly irregular follow-up visits and, eventually, discontinuation of maintenance care for approximately three years.

**Figure 5:**
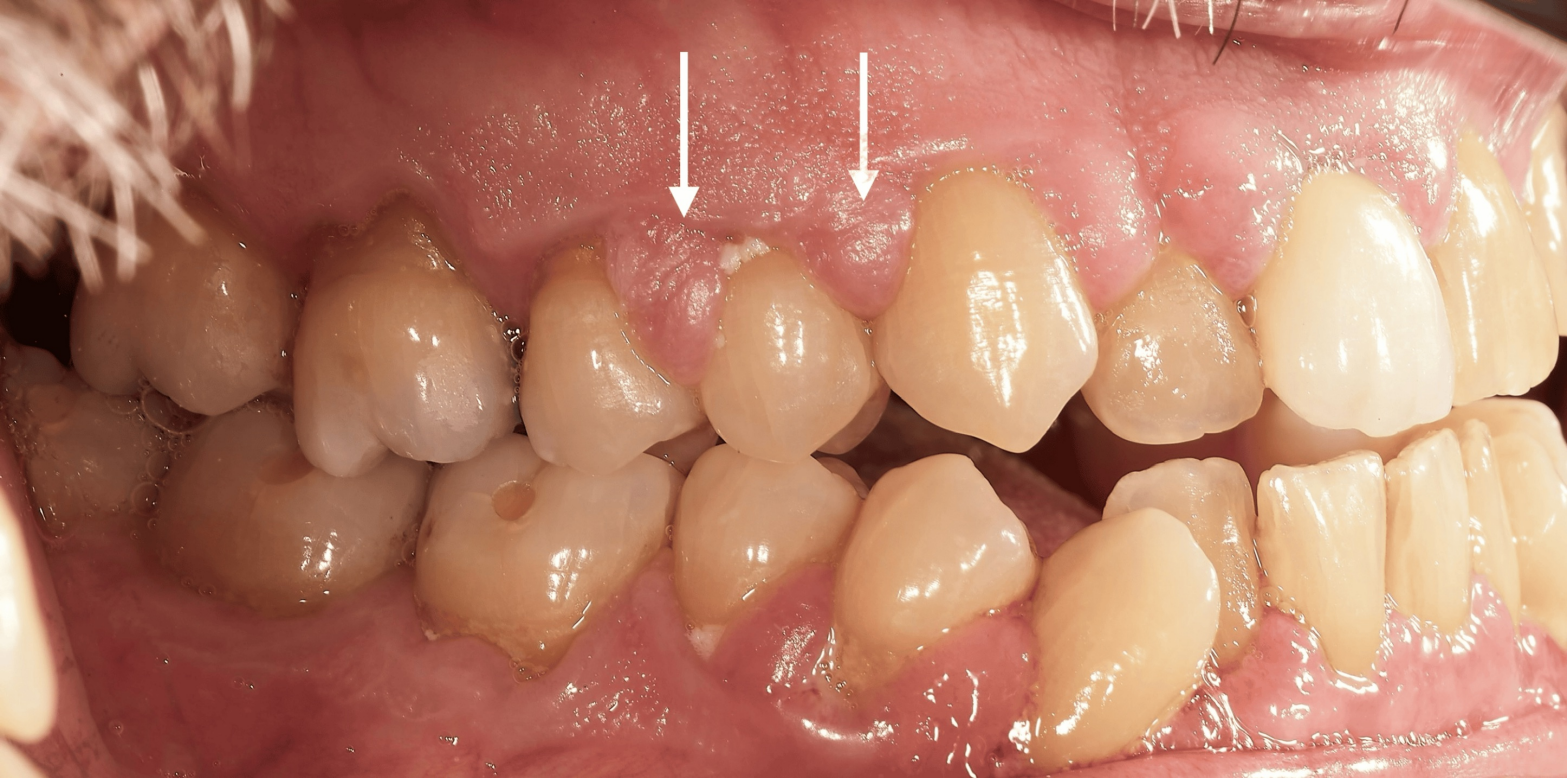
Intraoral photography (right lateral view) taken in 2025, showing lobulated gingival overgrowth with persistent hypertrophy of the interdental papillae and signs of inflammation (white arrows), consistent with disease progression in the absence of regular periodontal maintenance.

**Figure 6:**
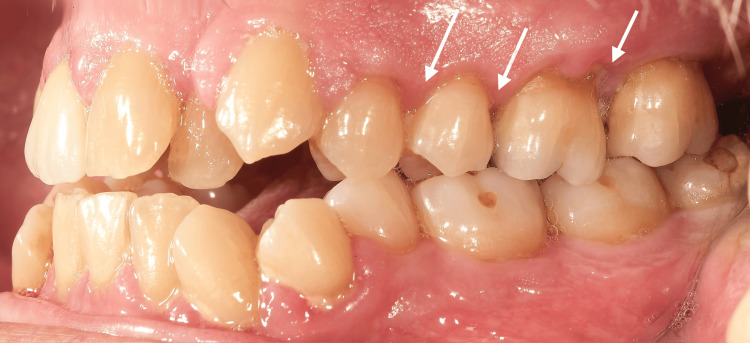
Intraoral photography (left lateral view) captured in 2025 reveals a clear regression of gingival overgrowth in selected papillary regions (white arrows), compared to images taken 23 years earlier. Despite this improvement, notable plaque accumulation persists on the posterior teeth, accompanied by bleeding on probing, periodontal pocket depths up to 5 mm, and clinical attachment loss ranging from 3 to 4 mm in specific sites.

**Figure 7:**
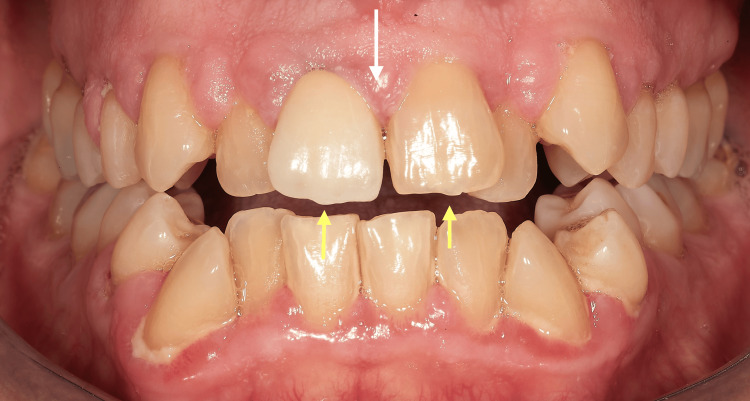
Intraoral frontal view captured in 2025, highlighting the flaring (splaying) of the maxillary central incisors (yellow arrows) and the presence of inflamed, hypertrophic papillary tissue (white arrow) between the central incisors.

**Figure 8:**
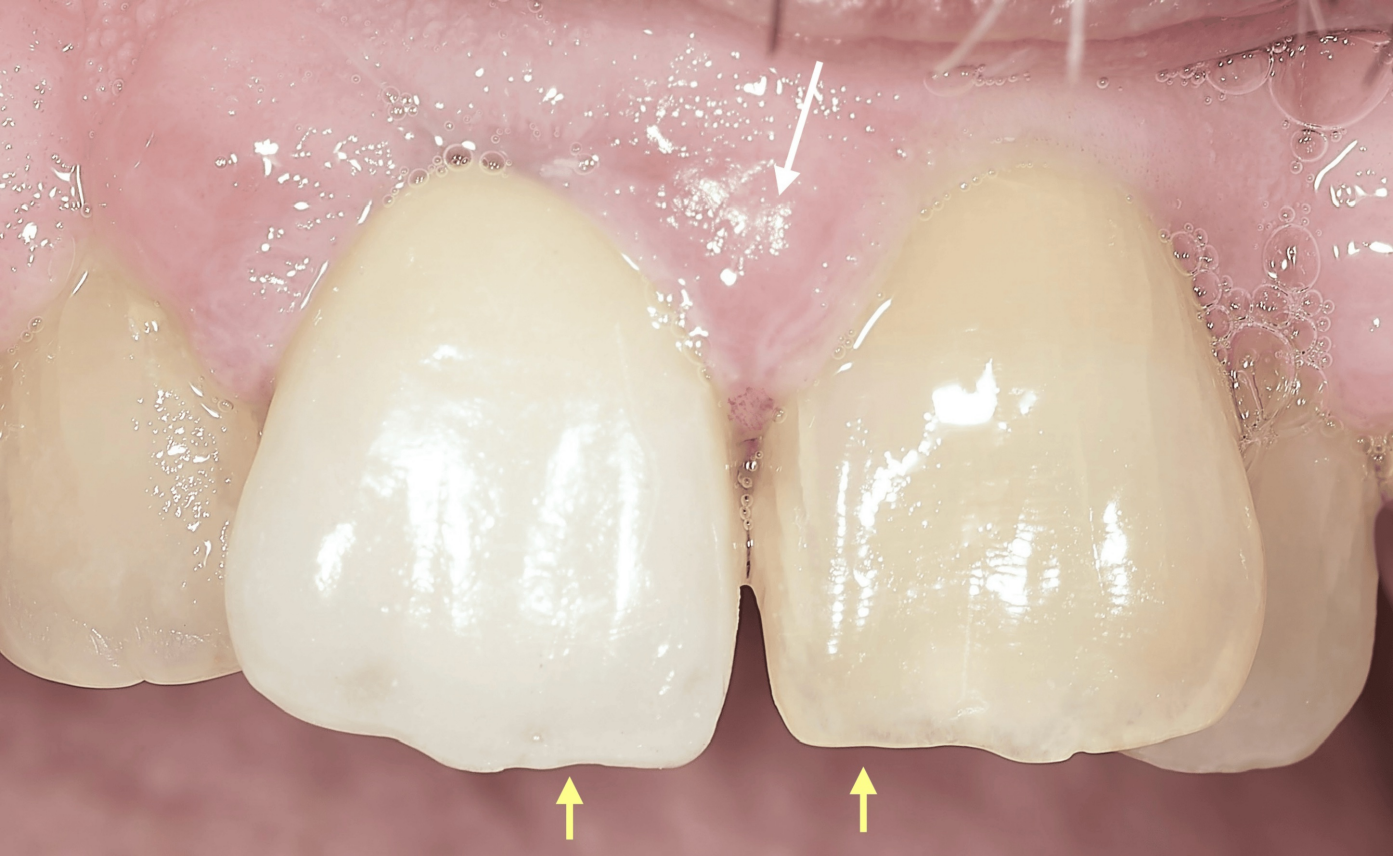
An intraoral frontal view captured in 2025 reveals flaring (splaying) of the maxillary central incisors (yellow arrows), inflamed and hypertrophic papillary tissue between the central incisors (white arrow), purulent exudate along the gingival margin, and generalized tooth discoloration with darker shades affecting the dentition.

In 2025, 23 years after the initial treatment, the patient returned to our clinic for re-evaluation. By that time, the periodontal condition had significantly deteriorated, reflecting the cumulative effects of plaque accumulation, gingival overgrowth, irregular attendance, and prolonged absence of professional monitoring. Clinical examination revealed a marked worsening consistent with localized Stage II, Grade B periodontitis, affecting less than 30% of the dentition, with a maximum probing depth of ≤5 mm and predominantly horizontal bone loss. Notably, flaring of the maxillary central incisors was observed, accompanied by purulent exudate along the gingival margin, generalized tooth discoloration, and bleeding on probing across multiple sites (Figures 5-8).

These findings suggest that the prolonged interruption of supportive periodontal care may have contributed to biofilm re-accumulation, sustained inflammatory activity, and gradual periodontal deterioration. They underscore the critical importance of continuous periodontal monitoring, especially in patients with a history of cardiac transplantation. The patient reported that the progressive gingival enlargement primarily affected his self-confidence and comfort during speaking and eating, rather than causing pain. His main concern was aesthetic and functional impairment, particularly the displacement of the maxillary central incisors.

Consequently, nonsurgical periodontal therapy was re-initiated. The treatment consisted of a single session of full-mouth scaling and root planing under local anesthesia, complemented by mechanical debridement and meticulous polishing of all accessible root surfaces. No pharmacologic or surgical interventions were performed. Cold-blade gingivectomy and gingivoplasty were proposed but ultimately declined by the patient, who preferred a conservative, non-invasive approach. Supportive periodontal therapy was then scheduled at three-month intervals to monitor plaque control, gingival inflammation, and pocket stability.

To achieve a comprehensive characterization of the oral microbiota, DNA-based metagenomic sequencing was performed on samples obtained from both the dorsal surface of the tongue and the subgingival plaque between the maxillary central incisors (Genomica S.r.l., Rome, Italy). Genomic DNA was isolated from the biological sample. Subsequently, a comprehensive oral microbiome profile was obtained through an advanced DNA sequencing technology known as Next Generation Sequencing (NGS), utilizing barcoded sequencing of the seven hypervariable regions (V2, V3, V4, V6, V7, V8, and V9) of the bacterial 16S ribosomal RNA (rRNA) gene. This gene, conserved across all bacterial species, contains variable regions with species-specific DNA sequences. These features enable precise taxonomic assignment and relative quantification of each bacterial species present in the sample. The interdental site was selected for analysis due to exhibiting the greatest attachment loss and the most pronounced inflammatory signs, including edema, papillary hypertrophy, purulent exudate along the gingival margin, and bleeding on probing. In accordance with microbiological sampling protocols, the patient was advised to avoid antibiotic intake as well as the use of chlorhexidine mouthrinses and chlorhexidine gel for at least one week prior to specimen collection.

**Figure 9:**
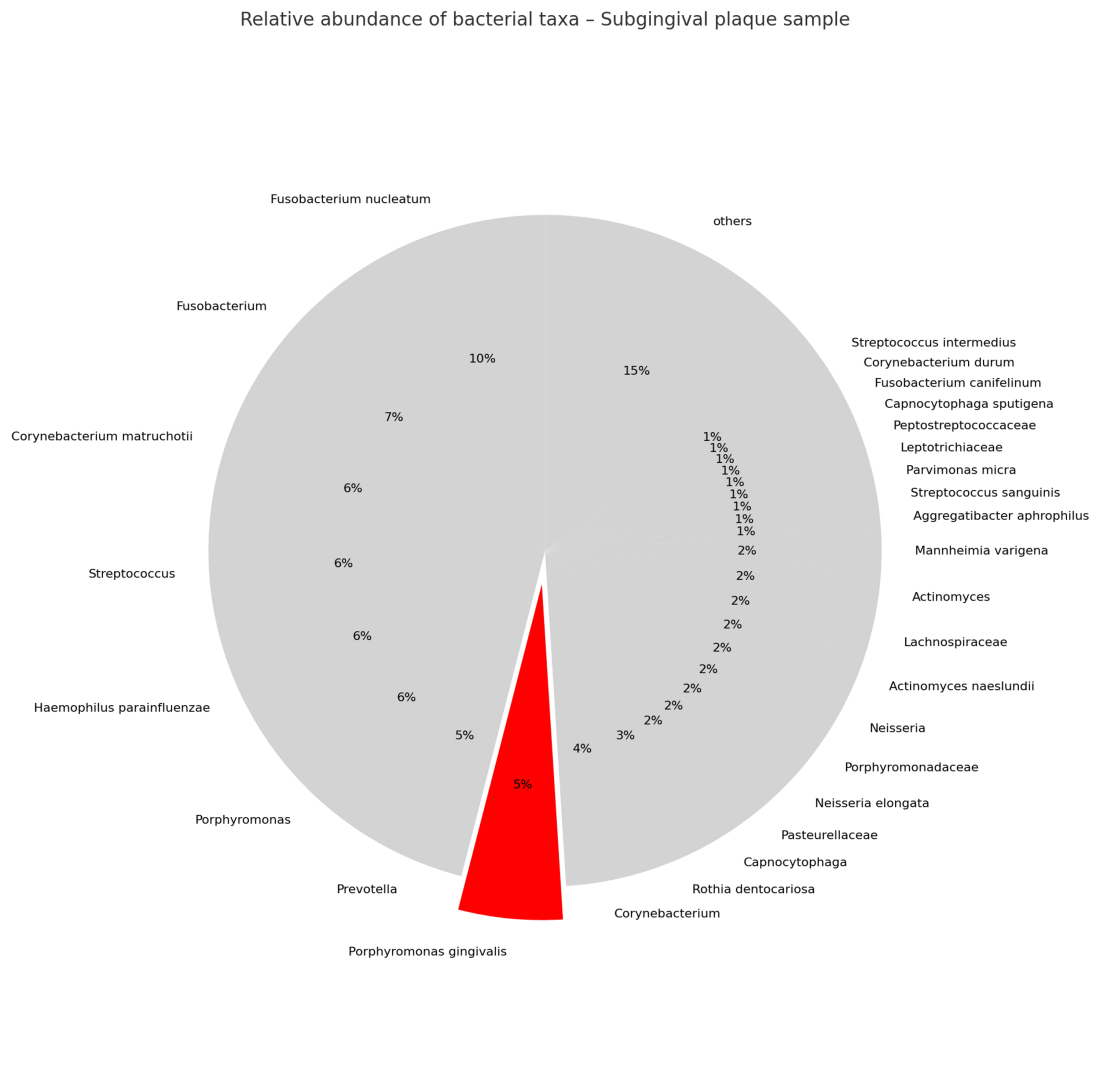
Relative abundance of bacterial taxa identified in the subgingival plaque sample from the maxillary central incisors pocket, collected during the 2025 follow-up visit, revealing a dysbiotic microbial profile. The red-colored slice represents the relative abundance of Porphyromonas gingivalis, a keystone pathogen implicated in the onset and progression of periodontal disease. Of particular note, the detection of Fusobacterium nucleatum highlights its ecological role as a bridging organism contributing to subgingival biofilm maturation and dysbiosis. Figure created by the authors using open-source software (Python/matplotlib).

Microbiological analysis revealed that the subgingival plaque microbiota was dominated by *Fusobacterium nucleatum* (10%), *Fusobacterium* (7%), *Corynebacterium matruchotii* (6%), *Streptococcus* (6%), *Haemophilus parainfluenzae* (6%), *Porphyromonas* (6%), *Prevotella* (5%), *Porphyromonas gingivalis* (5%), *Corynebacterium* (4%), *Rothia dentocariosa* (3%), *Capnocytophaga* (2%), *Pasteurellaceae* (2%), *Neisseria elongata* (2%), *Porphyromonadaceae* (2%), *Neisseria* (2%), *Actinomyces naeslundii* (2%), *Lachnospiraceae* (2%), *Actinomyces* (2%), *Mannheimia varigena* (2%), *Aggregatibacter aphrophilus* (1%), *Streptococcus sanguinis* (1%), *Parvimonas micra* (1%), *Leptotrichiaceae* (1%), *Peptostreptococcaceae* (1%), *Capnocytophaga sputigena* (1%), *Fusobacterium canifelinum* (1%), *Corynebacterium durum* (1%), *Streptococcus intermedius* (1%), others (15%) (Figure 9).

**Figure 10:**
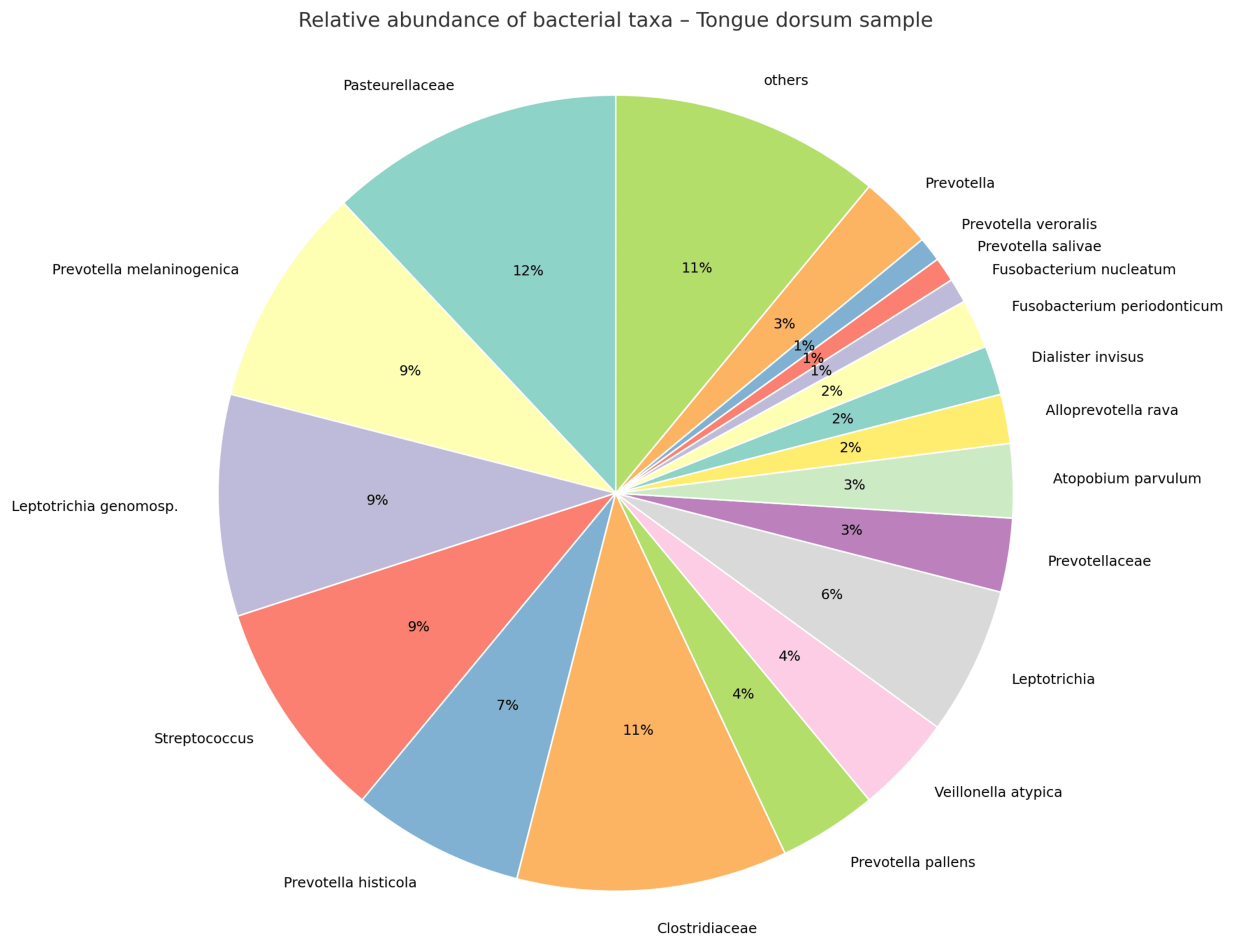
Relative abundance of bacterial taxa identified in the tongue dorsum sample, collected during the 2025 follow-up visit, showing a microbial composition consistent with relative eubiosis. Figure created by the authors using open-source software (Python/matplotlib).

The tongue dorsum swab showed a different microbial distribution, with a higher representation of commensals such as *Pasteurellaceae* (12%), *Prevotella melaninogenica *(9%), *Leptotrichia genomosp.* (9%), *Streptococcus* (9%), *Prevotella histicola* (7%), *Clostridiaceae* (11%), *Prevotella pallens* (4%), *Veillonella atypica* (4%), *Leptotrichia* (6%), *Prevotellaceae* (3%), *Atopobium parvulum* (3%), *Alloprevotella rava* (2%), *Dialister invisus* (2%), *Fusobacterium periodonticum* (2%), *Fusobacterium nucleatum* (1%), *Prevotella salivae *(1%), *Prevotella veroralis* (1%), *Prevotella* (3%), and others (11%) (Figure 10).

Additionally, molecular analysis targeting the ITS1 region of the *Candida albicans* genome was conducted using real-time polymerase chain reaction (RT-PCR). Interestingly, no opportunistic organisms, such as Candida spp., were detected despite the patient's immunosuppressed condition, a finding that contrasts with their commonly observed colonization in immunocompromised hosts. This dual-site sampling strategy enabled a broader assessment of microbial dysbiosis associated with DIGO and underlying periodontal pathology. Notably, the patient demonstrated improved adherence to maintenance visits following discussion of his metagenomic results, which increased his awareness of the microbial basis of disease recurrence.

The 2025 re-intervention resulted in a marked reduction of bleeding on probing and gingival inflammation, as well as an improvement in oral hygiene. While probing depths remained stable in the most affected areas, the overall clinical condition clearly improved. The patient is currently under supportive periodontal therapy, with recall visits scheduled every three months to maintain long-term stability. Future follow-ups will allow continued observation of both gingival overgrowth dynamics and periodontal status, providing valuable longitudinal data on the relationship between immunosuppressive therapy, microbiota composition, and clinical response.

## Discussion

DIGO is a well-documented adverse effect of immunosuppressive and calcium channel-blocking agents, particularly in transplant recipients maintained on cyclosporine-based regimens [10]. The clinical case presented here illustrates a typical manifestation of DIGO in a long-term cardiac transplant patient, complicated by persistent biofilm accumulation and moderate periodontitis. Although surgical management of gingival overgrowth has been recommended by several authors [11], in this case, the condition, though severe enough to compromise oral hygiene, function, and aesthetics, was treated exclusively through non-surgical periodontal therapy, in accordance with the patient’s decision to decline surgical intervention. In this case, the medical regimen could not be modified; therefore, the therapeutic approach relied entirely on non-surgical periodontal intervention and supportive periodontal therapy.

At the 23-year follow-up, a microbiological assessment of the tongue dorsum and subgingival biofilm was conducted using DNA-based techniques. No such investigation had been performed at baseline. This diagnostic adjunct aimed to better characterize the oral microbiota in a DIGO patient and monitor microbial shifts over time. Although this approach is not yet standard in routine periodontal management, it may provide useful insights, especially in complex cases involving immunocompromised individuals. An important and novel aspect of this case, only marginally documented in the literature, is the positive behavioral impact of metagenomic testing. The visualization of the patient's bacterial profile served as a powerful educational and motivational tool, leading to a significant increase in treatment adherence and home care compliance.

The coexistence of subgingival dysbiosis and tongue eubiosis suggests a site-specific ecological imbalance rather than a generalized oral infection, consistent with modern ecological plaque hypotheses. This finding supports the concept that periodontal disease progression arises from localized microenvironmental changes, driven by inflammation, altered nutrient gradients, and host immune modulation, rather than from uniform shifts across the entire oral ecosystem. In this context, it is well established that bacterial species within subgingival plaque tend to co-aggregate in specific microbial communities, commonly referred to as "complexes." Socransky et al. identified five major complexes, among which the so-called "Red Complex," composed of *Porphyromonas gingivalis*, *Tannerella forsythia*, and *Treponema denticola*, showed the strongest association with periodontal pocket depth and bleeding on probing [12]. In the present case, although *Porphyromonas gingivalis* represented only 5% of the total bacterial abundance, its detection remains clinically relevant given its keystone pathogen role and its capacity to disrupt host-microbe homeostasis even at low concentrations.

The pathogenesis of DIGO is multifactorial. Besides the direct pharmacological effects on gingival fibroblasts, local inflammation and dental plaque appear to modulate the severity of overgrowth [13]. Evidence suggests that elevated tissue levels of transforming growth factor β1 (TGF-β1), as seen in cyclosporine-treated patients, may promote extracellular matrix accumulation and fibrosis [14]. Effective plaque control and periodontal maintenance are therefore essential components of DIGO management [15].

Cao et al. emphasized that a prerequisite dental evaluation is typically recommended for patients following organ transplantation, as lifelong immunosuppression may increase susceptibility to infection spread. Their cross-sectional study revealed that periodontal health status was significantly associated with a history of heart transplantation among Chinese adults, with transplant recipients exhibiting worse periodontal conditions compared to matched controls. These findings underscore the importance of integrating oral health assessments into the pre- and post-transplant care protocols to mitigate systemic complications [16].

Interestingly, systemic inflammation in heart transplant recipients has also been associated with periodontal status. Sezgin et al. reported that elevated levels of serum high-sensitivity C-reactive protein (hs-CRP) in cardiac transplant patients were significantly correlated with the presence and severity of periodontitis [17]. These findings further support the integration of regular periodontal evaluations in post-transplant care, not only to address oral complications but also as part of systemic inflammatory control. Moreover, numerous studies have underscored the link between periodontal disease, atherosclerosis, and cardiovascular morbidity, reinforcing the importance of routine oral health monitoring in medically vulnerable populations. In particular, non-surgical periodontal therapy has demonstrated efficacy not only in improving clinical periodontal parameters but also in modulating systemic inflammation, evidenced by reductions in plasma levels of interleukin-6 (IL-6), C-reactive protein (CRP), and fibrinogen. These findings suggest that periodontal interventions may contribute to a broader cardiovascular risk mitigation strategy, especially in patients with predisposing systemic conditions [18].

This case reinforces the importance of a multidisciplinary approach in transplant recipients presenting with gingival enlargement. Coordination between dental and medical teams is crucial to ensure safe and effective treatment, even when systemic therapy cannot be altered, and evidence regarding the efficacy of nonsurgical periodontal treatment in reducing gingival overgrowth induced by cyclosporine-A [19]. Furthermore, histopathological and microbiological assessments may add diagnostic value, particularly in differentiating DIGO from neoplastic or infectious lesions in immunosuppressed patients [20,21].

Limitations

This report has several limitations that should be acknowledged. First, as a single case report, the findings cannot be generalized but rather serve to illustrate a long-term clinical scenario under cyclosporine therapy. Second, baseline microbiological data were not available, limiting a direct longitudinal comparison of microbial composition over time. Third, the cyclosporine regimen could not be modified due to the patient's medical status, which may have influenced the persistence of gingival enlargement. Finally, the patient's refusal of surgical intervention restricted the therapeutic options to non-surgical management, which, although effective in maintaining acceptable periodontal stability, did not allow for full resolution of gingival overgrowth.

## Conclusions

This single case provides a rare 23-year longitudinal perspective on cyclosporine-induced gingival overgrowth in a heart transplant recipient. The clinical course showed alternating phases of regression and recurrence, illustrating the potential importance of continuous periodontal maintenance in immunosuppressed patients. DNA-based metagenomic analysis offered additional descriptive insight into the microbial profile and, while exploratory, may suggest avenues for individualized preventive strategies. Nevertheless, these observations must be interpreted with caution. The conclusions are limited by the single‑case design, the absence of baseline microbiological data, the lack of cyclosporine serum levels, and the qualitative nature of the clinical outcomes. Any inference regarding the motivational role of metagenomic testing or the causal relationship between interruption of maintenance and DIGO recurrence should be considered speculative. Further longitudinal, controlled studies are warranted to better delineate the interplay between systemic drug levels, local inflammatory burden, and patient compliance in shaping disease trajectory.
